# Scoping review of values elicitation tools for treatment decisions in hepatocellular carcinoma

**DOI:** 10.1186/s12876-024-03167-1

**Published:** 2024-02-28

**Authors:** Gabrielle Ritaccio, A. Sidney Barritt IV, Jamie L. Conklin, Daniel R. Richardson, Donna M. Evon, Hanna K. Sanoff, Ethan Basch, Stephanie B. Wheeler, Andrew M. Moon

**Affiliations:** 1 Division of Gastroenterology and Hepatology, University of North Carolina at Chapel Hill School of Medicine, 8009 Burnett Womack Bldg, CB#7584, Chapel Hill, NC 27599-7584 USA; 2 UNC Liver Center, University of North Carolina at Chapel Hill School of Medicine, Chapel Hill, NC USA; 3 Health Sciences Library, University of North Carolina at Chapel Hill, Chapel Hill, NC USA; 4 Division of Hematology, Department of Medicine, University of North Carolina at Chapel Hill School of Medicine, Chapel Hill, NC USA; 5 Lineberger Comprehensive Cancer Center, University of North Carolina, Chapel Hill, NC USA; 6 Division of Oncology, Department of Medicine, University of North Carolina at Chapel Hill School of Medicine, Chapel Hill, NC USA; 7 Center for Health Promotion and Disease Prevention, University of North Carolina at Chapel Hill, Chapel Hill, NC USA; 8 Department of Health Policy and Management, Gillings School of Global Public Health, University of North Carolina at Chapel Hill, Chapel Hill, NC USA

**Keywords:** Hepatocellular carcinoma, Discrete choice experiment, Decision aids, Shared decision making, Probability trade-off

## Abstract

**Background:**

Treatment choices in hepatocellular carcinoma (HCC) involve consideration of tradeoffs between the benefits, toxicities, inconvenience, and costs. Stated preference elicitation methods have been used in the medical field to help evaluate complex treatment decision-making. The aim of this study was to conduct a scoping review to assess the evidence base for the use of preference elicitation tools or willingness to pay/willingness to accept methods for HCC treatment decision-making from both the patient and provider perspective.

**Methods:**

We performed a scoping review to identify abstracts or manuscripts focused on the role preference elicitation tools or willingness to pay/willingness to accept methods for HCC treatment options among patients, caregivers, and/or providers. Two researchers independently screened full-text references and resolved conflicts through discussion. We summarized key findings, including the type and setting of preference-elicitation tools used for HCC treatment decisions.

**Results:**

Ten published abstracts or manuscripts evaluated the role of preference elicitation tools for HCC treatments. The studies revealed several attributes that are considered by patients and providers making HCC treatment decisions. Many of the studies reviewed suggested that while patients place the most value on extending their overall survival, they are willing to forgo overall survival to avoid risks of treatments and maintain quality of life. Studies of physicians and surgeons found that provider preferences are dependent on patient characteristics, provider specialty, and surgeon or hospital-related factors.

**Conclusion:**

This scoping review explored both patient and physician preferences towards treatment modalities in all stages of HCC. The studies revealed a large scope of potential attributes that may be important to patients and that many patients are willing to forgo survival to maintain quality of life. Further research should explore both preference elicitation of currently available and emerging therapies for HCC as well as the use of this data to develop patient-facing tools to assist in navigating treatment options.

**Supplementary Information:**

The online version contains supplementary material available at 10.1186/s12876-024-03167-1.

## Background

Hepatocellular carcinoma (HCC) is the most common type of primary liver cancer and deaths due to HCC are increasing in the US [1]. There are a number of treatment options for HCC including surgical resection, liver transplantation, locoregional therapies and systemic therapies [2]. Treatment options for HCC have improved dramatically in recent years. Emerging locoregional options include external beam radiation therapy (EBRT), drug-eluting bead (DEB)-TACE, radiation segmentectomy (i.e. highly selective form of radioembolization) and locoregional therapy combinations [3]. In a short period, there has been a dramatic increase in systemic therapies [4–11], including combination immunotherapy as the new first-line option for advanced HCC [5]. The potential synergistic mechanisms between locoregional therapy and systemic treatments has led to trials exploring combined locoregional/systemic therapies [4, 12]. These new combinations present the possibility for improved progression-free survival but may also be associated with greater risks of treatment-related toxicities and financial costs.

There are some signs that the mortality rate from HCC is beginning to decline in the US and other countries due to multiple factors including availability of treatments for viral hepatitis, surveillance for HCC leading to earlier detection, and improvements to HCC treatments. The consideration of patient preferences into all aspects of HCC care may contribute to the improving prognosis of HCC by increasing patient satisfaction and participation in care [13–15]. In light of the complexity of treatment decisions in HCC, multidisciplinary models have emerged [16] including multidisciplinary tumor boards and clinics. Treatment choices involve consideration of tradeoffs between the benefits, toxicities, inconvenience, and/or costs of therapeutic options. Values elicitation tools, such as discrete choice experiments (DCE) and conjoint analysis, are stated preference elicitation methods with mathematical frameworks that have been used in the medical field to evaluate complex treatment decision-making from both the patient and provider perspective. Values elicitation tools including DCEs have been studies extensively in other oncologic populations but their use in HCC has not been summarized [17]. Improved understanding of the role of values elicitation tools in HCC treatment decisions would help guide efforts to incorporate such tools into the evolving, increasingly complex treatment landscape.

The aim of this study was to conduct a scoping review of preference elicitation tools or willingness to pay/willingness to accept methods relating to HCC treatments with a goal to assess the extent of available evidence for the use of these paradigms to systematically study health care decision-making in this clinical scenario.

## Methods

### Eligibility criteria

We performed a scoping review including studies that (1) assessed the role of preference elicitation tools or willingness to pay/willingness to accept methods (“conjoint analysis”, “discrete-choice experiments”, “stated-choice methods”, “stated preference”, “contingent valuation”, “willingness to pay”, “willingness to accept”, “trade-off”, “standard gamble”) for treatment options for hepatocellular carcinoma and (2) included a study population of patients, caregivers and/or providers. Studies were excluded if they were non-English language.

### Information sources and search strategy

A health sciences librarian searched the following four databases from their dates of inception through the last search date of August 18, 2022: PubMed, the Cochrane Library, Embase (Elsevier), and Scopus (Elsevier). The search strategy included both subject headings and keywords, including a variety of synonyms, for the two main concepts of hepatocellular cancer and patient preferences. The search contained no filters or limits. The complete, reproducible search strategy for all databases is available in the appendix (Appendix A). All references were exported to Endnote X9 (Philadelphia, Pennsylvania, USA) to remove duplicates.

### Study selection

All unique references were placed into Covidence systematic review software (Veritas Health Innovation, Melbourne, Australia, available at www.covidence.org) to complete and track the study selection process. Two researchers first independently screened each reference’s title and abstract for eligibility criteria, and conflicts were resolved via consensus. Next, two researchers independently screened full-text references, and resolved conflicts through discussion. If references were identified in abstract form, a PubMed search was performed to evaluate whether a full text had been published in the interim.

### Data charting and synthesis

Data charting was completed independently by two researchers, and conflicts were resolved through discussion. No quality assessments were performed given that this was a scoping review. We followed the Preferred Reporting Items for Systematic Reviews and Meta-Analyses (PRISMA) extension for scoping reviews to complete and report our review [18].

## Results

**Fig. 1:**
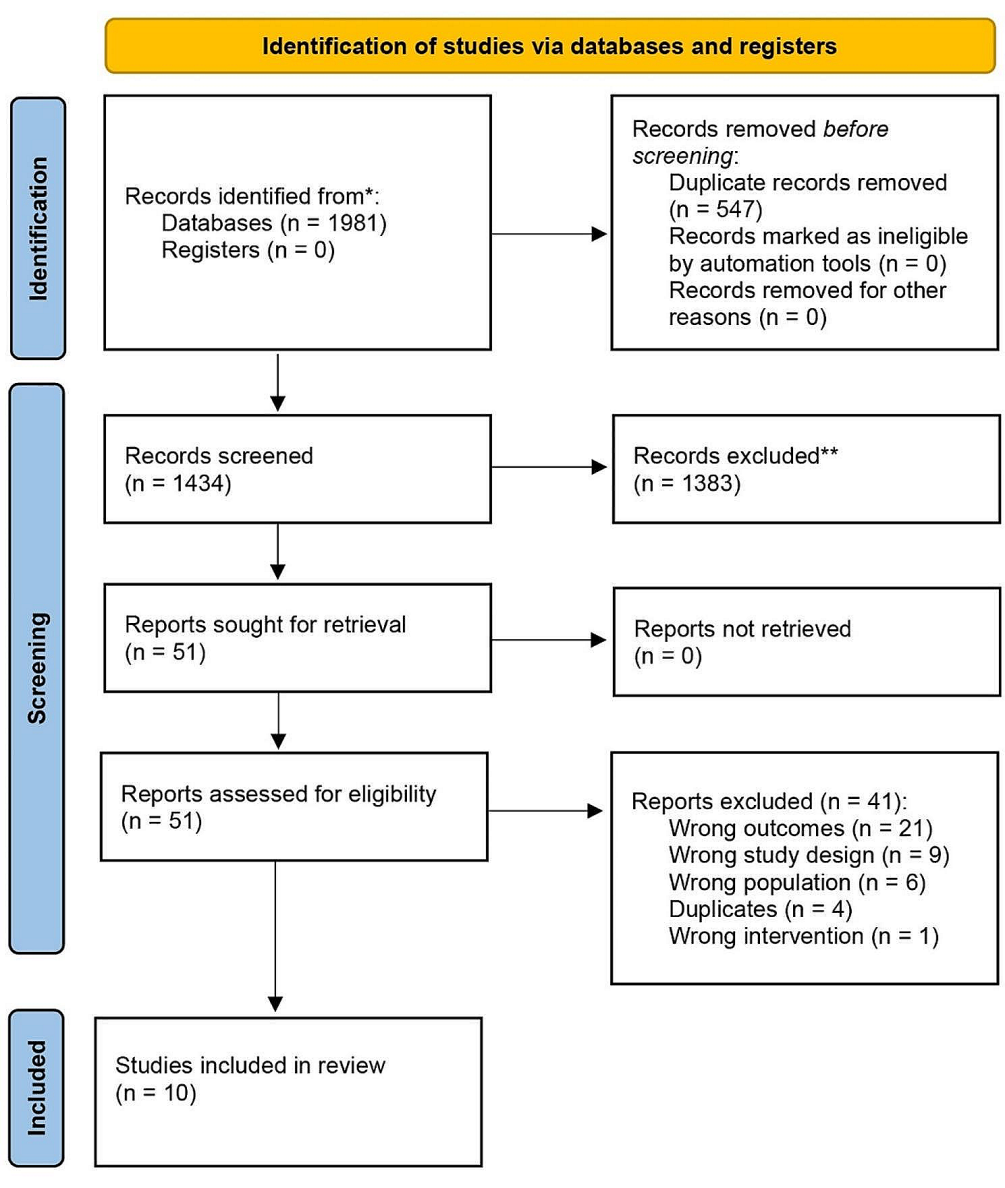
PRISMA diagram of included studies

The database searches returned 1981 results, and 1434 were screened against title and abstract (Fig. 1). After excluding 1383 studies, we assessed 51 studies for full-text eligibility. We excluded 41 of those based on wrong outcomes, study design, patient population, or intervention and ultimately included 10 studies in this review.

Study characteristics are included in Table 1. Included studies examined patient-preferences in early or advanced stage HCC (*n* = 5), a smartphone-based HCC treatment decision system (*n* = 2) and tradeoffs and treatment decisions based on surveys of surgeons (*n* = 2) and GI/hepatologists (*n* = 1). Three of 10 studies were industry funded.

**Table 1:** Characteristics of included manuscripts

Author	Setting	Population (n)	Primary Focus	Preference-elicitation tool	Key Results	Industry sponsorship
Chen (2012) [27]	Single-center (Taiwan)	53 “liver patients”	Use a multi-attribute utility theory to establish a decision model for liver cancer treatment selection and explore whether patients’ treatment preferences were concordant with their physicians’ recommendations	Web-based questionnaire	-Patients preferred active treatment in the case of advanced HCC -Factors impacting treatment decision most were cure rate, survival rate, and ability to provide self-care -Preferences differed significantly based on HCC stage and physician recommendations	N
Chiba (2019) [20]	Multi-center (Japan)	199 patients with HCC	Evaluate preferences for features associated with intermediate or advanced HCC treatments (sorafenib, TACE, and HAIC)	Web-based best-worst scaling, direct preference elicitation and willingness to try	-Oral medication perceived as most favorable -Risk of liver damages perceived as least favorable -Patients’ previous experience with treatment influence preferences regarding future treatments (i.e., likely favor the option they are most familiar with)	N
Li (2023) [21]	Agency recruited (US)	200 patients with self-reported unresectable HCC	Quantify patients’ benefit-risk preferences for attributes associated with first line systemic treatments for unresectable HCC (atezolizumab + bevacizumab vs. tyrosine kinase inhibitors)	Web-based discrete choice experiment	-Patients prioritized avoiding side effects (e.g., moderate-to-severe palmar-plantar syndrome and hypertension) that would severely impact their quality of life -Patients regarded an additional 10 months of maintaining daily function without decline to be as important or more important than an additional 10 months of overall survival	Y
Lo (2021) [23]	Agency recruited (France, Germany, Spain, UK)	150 patients with self-reported HCC	Understand patient preferences for characteristics of advanced HCC treatments	Web-based discrete choice experiment	-Patients placed most value on extending overall survival -Patients will forego some months of life to avoid side effects or risks	Y
Molinari (2014) [19]	Multi-center (Canada)	75 patients with cirrhosis (Child-Pugh class A or B)	Elicit preference between HR and RFA in early-stage HCC	In-person probability trade off technique	-Informed cirrhotic patients prefer RFA for the treatment of early-stage HCC	N
Nathan (2011) [24]	Agency recruited (US)	336 surgeons “with an interest in liver surgery”	Quantify the impact of clinical factors and surgeon specialty on surgical decision making in early HCC	Web-based conjoint analysis	-Surgeon specialty (i.e., whether or not the surgeon was involved in liver transplantation) was at least as important as clinical factors in determining preference for initial therapy	N
Nathan (2013) [25]	Agency recruited (US)	336 surgeons “with an interest in liver surgery”	Understand the effect of surgeon- and hospital-related factors on surgical decision-making in early HCC	Web-based conjoint analysis	-Surgeon practice type, annual HCC patient volume, and procedures performed for HCC had significant association with choice of therapy -Surgeon’s involvement in procedure performed for HCC remained the strongest predictor of choice of therapy	N
Nathan (2014) [26]	Agency recruited (US)	119 physicians who treat HCC	Quantify the impact of clinical factors on choice of therapy for early HCC by gastroenterologists and hepatologists	Web-based conjoint analysis	-Clinical factors (i.e., tumor number and size, type of resection required, MELD score, and platelet count) had the largest effect on choice of therapy -No physician-related factors studied had an impact on choice of therapy	N
Parikh (2023) [22]	Physician and agency recruited (US)	150 patients with unresectable or metastatic HCC who had progressed on, or were intolerant to, first-line sorafenib therapy	Determine patients’ preferences for mode of administration and risk of adverse events for regorafenib (4 tablets once daily) vs. ramucirumab (once in 2 weeks IV for 30/60 minutes)	Web-based modified threshold technique	-All else being equal, patients preferred daily tablets to every 2 week IV -In the context of associated adverse events, most patients preferred every 2 week IV	Y
Wang (2022) [28]	Single-center (China)	180 primary liver cancer patients	Explore the effects of a “Shared Decision Making Assistant” smartphone application on the decision-making of informed patients	“Shared Decision Making Assistant” smartphone application	-Patients using SDM Assistant” had significantly lower decision conflict scores than those in the control group -Scores of “regret of decision making” between the two groups had no statistical significance after 3 months	N

Nine studies focused on quantifying patient or physician preferences for HCC treatment. They all used surveys to offer hypothetical choice between treatment options. Patients surveyed included those with cirrhosis or HCC. Physicians and surgeons surveyed all had experience caring for patients with HCC.

### Patient-oriented preference elicitation

Five studies explored patient preferences for HCC treatment. One of these studies [19] was conducted in patients with very early or early-stage HCC and the others were in patients with unresectable HCC.

#### Probability trade-offs for early-stage HCC treatments

Molinari et al. [19] studied probability trade-offs between radiofrequency ablation (RFA) and liver resection for small HCCs < 3 cm and to assess the threshold for survival benefit, disease-free survival and perioperative morbidity and mortality. They included adult patients in Canada with Child Pugh class A or B cirrhosis. Of note, none of these patients had a diagnosis of HCC. Preferences were elicited by probability trade off interviews conducted in-person by the primary investigator or research coordinator. The probability trade off technique involves a participant placing themselves in the position of a hypothetical individual affected by early-stage HCC with the treatment options of RFA and resection, deciding between these options based on an educational session and determining the variables and thresholds that influenced the decision. Thresholds are determined by changing the probabilities for good or bad outcomes in a systematic way (e.g., changing likelihood of overall survival or toxicity) until the respondent’s initial preference switches.

The study enrolled 75 participants with cirrhosis but without HCC. When proposing a hypothetical scenario of being diagnosed with HCC amenable to RFA or resection, 70.3% preferred RFA. Among participants who initially preferred RFA, the thresholds that resulted in a preference change included resection improving 5-year survival ≥ 15% or 3-year disease free survival ≥ 10%. Participants preferring RFA also changed their preference if the median probability of complications after RFA was ≥ 8%.

#### Best/worst scaling and direct elicitation for intermediate/late-stage HCC treatments

Chiba et al. [20] studied preferences for intermediate to advanced stage HCC treatments but included patients with all stages of HCC. They surveyed adult (> 20 years old) HCC patients in Japan using a cross-sectional online survey. They used best-worst scaling to prioritize 13 treatment features representing key differentiating characteristics of transarterial chemoembolization (TACE), hepatic artery infusion chemotherapy (HAIC), and sorafenib, an oral tyrosine kinase inhibitor. They also included direct preference elicitation to assess preference for TACE, HAIC and sorafenib based on standardized descriptions and assessed the likelihood that respondents would try a medication if it cost nothing but resulted in worsening of symptoms. Lastly, they assessed patients’ willingness to try an oral anti-cancer medication if it resulted in various delays in disease progression but resulted in severe hand-foot and skin reaction (HFSR).

The study included a total of 119 participants, of whom 41.2% had cirrhosis, 24.4% had early-stage HCC and 75.6% had intermediate or advanced stage HCC. All patients had received some HCC treatment with TACE, oral systemic therapy, HAIC and/or hepatic resection. In the best-worst scaling, the most favorable scores were given to the following attributes: oral medication taken twice a day, artery branches in liver are plugged by therapy, and treatment that prevents formation of new blood vessels that a cancer needs to grow. The least favorable features of a treatment were risk of liver damage that may prevent future cancer treatments, risk of complications due to implanting catheter and risk of stopping treatment due to side effects. The average likelihood of trying oral medication was 59.1% compared to 52.2% for TACE and 34.5% for HAIC (*p* < 0.001 for both pairwise comparisons). They reported that patients with sorafenib- or TACE-experienced preferred what they had received (60% and 65% respectively), however HAIC patients preferred alternative treatment options (90%). They also found that the mean maximum acceptable risk estimates for severe HFSR that patients were willing to accept for 3-, 6-, and 12-month delays in time to progression were 26.1%, 33.5%, and 45.5%, respectively.

#### DCEs and modified threshold techniques of treatments for advanced HCC

There have been three published manuscripts using web-based DCEs to assess preferences for treatments of advanced HCC [21–23]. All three of these studies were industry-sponsored, including by the manufacturers of atezolizumab/bevacizumab (Genentech) [21], ramucirumab (Eli Lilly) [22], and yttrium-90 microspheres (Sirtex) [23].

Li et al. [21] surveyed patients with self-reported unresectable HCC in the US using a cross-sectional web-based DCE survey. The survey included questions offering a hypothetical choice between treatment profiles defined by six attributes, each with three levels: overall survival, number of months to maintain daily function, severity of palmar-plantar syndrome, severity of hypertension, risk of gastrointestinal bleeding, and mode and frequency of administration. They enrolled 200 respondents with self-reported unresectable HCC, including a majority currently receiving treatment (85.5%). The highest importance was placed on moderate-to-severe palmar-plantar syndrome and moderate-to-severe hypertension, followed by an additional 10 months of maintaining daily function and additional 10 months of overall survival. The researchers also assess the minimal overall survival required to offset changes in other treatment attributes. Respondents stated they would require an additional 10 months of overall survival to accept any of the following: a decrease in the number of months one is able to perform activities without decline from 13 to 3 months, worsening palmar-plantar syndrome from no/mild to moderate/severe, or a worsening in hypertension from no/mild to moderate/severe. In the simulation exercise, the medication that was most preferred was an IV infusion administered every 3 weeks with a 7% chance of gastrointestinal bleeding, moderate-to-severe hypertension, no palmar-plantar syndrome, 13 months to maintain daily function, and 20 months of overall survival.

Parikh et al. [22] used an online modified threshold technique to assess tradeoffs between daily tablets and biweekly infusions in second-line treatment of unresectable HCC. They surveyed 150 participants in the US with unresectable or metastatic HCC who had progressed on or were intolerant to sorafenib treatment. Using a modified threshold technique design, they asked respondents to choose between regorafenib vs. ramucirumab which have similar efficacy and safety profiles, but different route of administration. Threshold questions factored in the seven clinically relevant factors that differentiate the two agents, including adverse events more common in regorafenib (hypertension, decreased appetite, HFSR, diarrhea) and those more common with ramucirumab (ascites, proteinuria, peripheral edema). Most patients preferred daily tablets (61.3%). However, when all risks were considered, 76.7% preferred IV infusion similar to ramucirumab and the utility gained of taking oral tablets instead of IV infusions was more than offset by the adverse event profile. For those who initially preferred oral tablets, the risk threshold differences that made them indifferent between tablets and IV infusion were 7.1% for hypertension, 7.9% for decreased appetite, 9.8% for hand-foot skin reaction and 6.8% for diarrhea.

Lo et al. [23] studied patient preferences for advanced HCC treatments using a DCE. They surveyed patients in the United Kingdom, Germany, Spain, and France. They included a total of 150 participants with HCC, including 20.7% with very early/early/intermediate stage, 43.3% with advanced stage and 36.0% with end-stage HCC. They considered 17 possible attributes and, after semi structured interviews and stakeholder feedback, included the following attributes: overall survival, treatment waiting time, mode of administration/treatment schedule, high blood pressure, nausea/vomiting/loss of appetite, fatigue/tiredness, diarrhea, and skin irritation. Overall survival was the most important attribute; respondents were 26% more likely to prefer a treatment for each additional month of overall survival. Patients were 8% less likely to prefer a treatment for each additional week of waiting and, all attributes held constant, respondents were 22% less likely to choose IV therapy compared to liver directed treatment such as radioembolization. Patients were willing to trade overall survival to reduce adverse event risks: reducing risk of hypertension by 10% was equivalent to trading 1.6 months of overall survival; reducing risk of diarrhea, skin irritation, or gastrointestinal side effects by 10% was equivalent to trading 1.0 month of overall survival; to avoid a 50% of fatigue, patients were willing to trade 4.0 months of overall survival.

### Preference elicitation studies of HCC treatments involving physicians

Three studies, all conducted by Nathan et al., [24–26] explored physician preferences for initial HCC treatment.

One study explored surgical decision-making and used conjoint analysis to quantify the relative impact of clinical factors on choice of therapy as well as the impact of surgical specialty on decision-making [24]. They performed an online survey, with responses from 336 surgeons with an interest in liver surgery. They found that choice of therapy varied widely but overall, liver transplant and resection were preferred equally. They also found that the surgeon specialty (i.e., whether the surgeon was involved in liver transplantation) played a role that was at least as important as clinical factors in determining preference for initial therapy. When asked their general preferences for initial surgery for HCC, non-transplant surgeons were significantly more likely to recommend resection over transplant (50% resection vs. 41% transplant) compared with transplant surgeons (31% resection vs. 63% transplant) (*p* < 0.001). However, when the weight of each clinical factor was allowed to vary by surgeon specialty, the residual independent effect of surgeon specialty on the decision between resection and transplant was negligible. The clinical factors that had the largest effects on the choice of therapy included the type of resection required, tumor number and size, and platelet count. The need for a major resection, multifocal disease, and low platelet count all increased the preference for liver transplant.

In a separate publication, Nathan et al. assessed the influence of non-clinical factors (i.e., surgeon or hospital-related factors) on initial treatment choice in patients with early-stage HCC [25]. These data were obtained from the same online sample of surgeons with an interest in liver surgery (*n* = 336). They used conjoint analysis to systemically study decision-making using case scenarios. They assessed nonclinical factors such as practice type, years in practice, fellowship training, training in liver transplant, annual HCC patient volume, whether resection, RFA, or liver transplant were performed at the hospital where they primarily worked, and whether the participant performed resection, RFA, transplant or a combination of these treatments. After adjustment for clinical factors, they reported that surgeon practice type, annual HCC patient volume, and procedures performed for HCC had significant association with choice of therapy. When analyzed in conjunction with surgeon-specific variables, the type of procedures performed for HCC remained the strongest predictor of choice of therapy. Transplant surgeons who did not also perform RFA were less likely than transplant surgeons who did offer RFA to choose RFA over transplant (relative risk ratio 0.38, *p* < 0.001). Non-LT surgeons were more likely than LT surgeons who also offered RFA to choose RFA over LT (relative risk ratio 2.24, 95% CI 1.34–3.74). They also reported that surgeons whose primary hospital performed liver transplant were more likely to choose transplant over resection and RFA, even if they themselves did not perform transplant (relative risk ratio 1.27 and 3.33, *p* < 0.001).

Nathan et al. applied a similar framework to explore clinical decision-making of gastroenterologists and hepatologists [26]. This online survey included 119 practicing physicians who had completed training and evaluate at least five HCC patients per year. They used conjoint analysis to quantify the relative impact of clinical factors on choice of therapy as well as the impact of physician-related factors. They found a slight majority of respondents preferred liver transplant over surgical resection (52% vs. 44%), similar preference for resection and RFA (43% vs. 44%) and preference for RFA over transplant (55% vs. 37%). Tumor number and size, type of resection required, MELD score, and platelet count had the largest effect on choice of therapy. No physician-related factors (practice type, years in practice, specialty, percent clinical time, or HCC volume) were associated with choice of therapy.

### Comparison of patient preferences and physician recommendations

Chen et al. [27] explored whether patients’ decisions were concordant with their physicians’ recommendations. They included 53 patients with liver disease, including 66% who had liver cancer from a single center in Taiwan. Through interviews with patients and providers, 13 factors were identified that were associated with treatment decision-making. These included physical and psychosocial considerations such as rate of curability, physician recommendation, and quality of life. They then designed a questionnaire of treatment-choice questions that took these factors into consideration and was stratified by liver cancer stages and in accordance with Barcelona Clinic Liver Cancer (BCLC) practice guidelines. They then used multi-attribute utility theory to establish a decision model for treatment selection. The factors that impacted patient treatment decisions most were cure rate, survival rate, and ability to provide self-care. They also found that patients with advanced stage disease still preferred active treatment. Interestingly, when patients’ treatment preferences were compared across different variables, significant differences were seen with respect to HCC stage and physician recommendations.

### The effect of a smartphone application on decision-making in HCC

Wang et al. [28] assessed whether a smartphone app with a built-in preference elicitation could assist patients in navigating the complex decision making associated with determining their HCC therapy. Chinese patients with primary liver cancer (*n* = 180) were randomized to a control group (*n* = 90) vs. intervention group (*n* = 90) who had access to the “Shared Decision Making” app. The primary outcome was patients’ perceptions of decision uncertainty measured using a decisional conflict scale. The app included two core parts: the treatment knowledge center and the decision aids path. The knowledge center included information on both primary liver cancer and the 12 treatment options offered including indications, contraindications, preoperative preparation, postoperative care, complications, advantages, disadvantages, subsequent therapy, postoperative recurrent rate, and health education after discharge. The decision aids path was based on the Ottawa decision support framework, which guides practitioners and researchers to assess participants’ decisional needs, provide decision support interventions, and evaluate the effects on decisional outcomes. It included five steps to help clarify the choice of preferred treatment options: (1) Meeting with physician to determine available treatment options (2) Using the app to compare alternative treatment options (3) Exploring the preferences and scoring the risks and benefits using a Likert 5-point scoring system through the app. (4) Knowledge testing to determine whether the participants’ choice was based on correct understanding of disease treatment knowledge, and (5) A physician meeting to finalize treatment plan. The app also encouraged patients to evaluate and summarize their decision the following day through the app.

Results showed significantly lower levels of decisional conflict in the intervention group (16.89 +/- 8.8) compared to the control group (26.75 +/-9.79; *p* < 0.05). Secondary outcomes included decision preparation, decision self-efficacy, satisfaction with decision-making, knowledge of treatment and decision regret. The intervention group also had significantly higher decision preparation, self-efficacy, and satisfaction scores compared to the control group.

### Summary of attributes identified

**Fig. 2:**
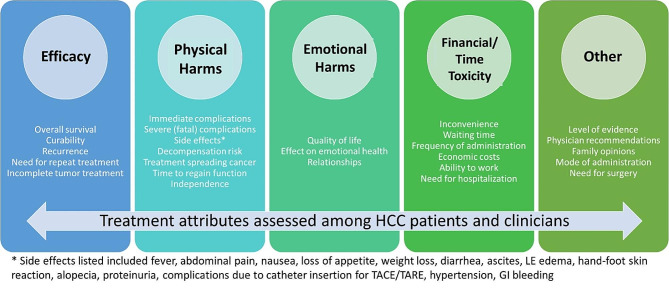
HCC treatment attributes considered by patients and clinicians

Attributes of treatments identified are shown in Fig. 2 with most focusing on efficacy, physical, and emotional harms. In the patient preference studies, efficacy was typically approximated by overall survival. Physical and emotional harms were often interdependent as preferences focused on reduced risk of severe side effects that would impact self-care and quality of life.

## Discussion

This review identified 10 published studies evaluating the role of preference elicitation tools for HCC treatment. The studies revealed a large scope of potential attributes that may be important to patients. A couple of studies suggest that patient preferences differ from physician recommendations.

This scoping review identified several gaps in the literature. The majority of these studies focused either on early [19] or advanced HCC [21–23]. The only study that considered treatments for intermediate stage HCC [20] was limited to assessing the tradeoffs between TACE, sorafenib and HAIC and therefore is not generalizable to current-day intermediate stage treatment options, which include transarterial radioembolization, external beam radiation therapy, and combination immunotherapy (atezolizumab/bevacizumab and durvalumab/tremelimumab). Much of the focus in advanced stage HCC was on oral systemic therapy, which is no longer a first-line treatment, now that combination immunotherapy has demonstrated superior overall survival and health-related quality of life [5, 29]. Furthermore, there was no assessment of emerging therapies including combinations of locoregional therapies plus immunotherapy. Understanding and reconciling patient and physician preferences for HCC treatment is imperative to delivering patient-centered evidence-based care. However, only one study [27] explored whether patients’ treatment preferences were concordant with their physicians’ recommendations. There were no studies that specifically evaluated willingness to pay for various treatments or assessment of direct and indirect costs of therapies. This is a notable omission in light of the fact that approximately 20% of cancer survivors experience financial toxicity, which is associated with impaired quality of life [30–32].

A Good Practices Report of the International Society of Pharmacoeconomics and Outcomes Research (ISPOR) generated a “roadmap” to guide studies of patient preferences in order to generate useful data for decision-makers [33]. This blueprint recommends that researchers account for the context for decisions, purpose of the study, the applicable population, study methods, and the impact of the preferences identified. Studies identified in this review did generally adhere to this framework. However, in these studies, the context was often hypothetical as patients themselves had risk factors for the development of the clinical question at hand (e.g., they carried a diagnosis of cirrhosis) but did not necessarily carry the diagnosis in question (e.g., advanced HCC). Future studies could consider eliciting preferences in real-time from patients actively making treatment decisions for their own care. With regards to impact, all preference elicitation studies sought to understand preferences for treatment with the aim of informing therapeutic decision-making. However, only one study [28] developed this into a patient-facing tool.

While well-validated preference-elicitation tools may assist with the incorporation of patient values into treatment choices, these preferences should be viewed as a supplement rather than a replacement of optimal patient selection based on tumor burden, liver function and performance status. Future studies should focus on how best to incorporate patient preferences into validated treatment algorithms such as the BCLC staging system [34]. In addition to informing treatment choice, preferences could also be used to guide additional support needed by HCC patients. For instance, patients who place a high priority on avoiding symptoms may benefit from earlier palliative care interventions that can be led by nurses, physicians, psychologists, or social workers. A recent pilot trial reported that earlier palliative care involvement improved health-related quality of life and symptoms in patients with HCC [35].

Findings of this study need to be interpreted in the context of potential limitations. This was a scoping review that did not involve extraction of meta-data or quality assessments of studies. However, given the significant heterogeneity of the studies included, direct comparisons were not feasible. Furthermore, we excluded non-English abstracts which may have led to the omission of potentially relevant manuscripts.

## Conclusion

This scoping review assessed the prior literature on the role of preference elicitation tools or willingness to pay/willingness to accept methods for treatment options for HCC. This review explored both patient and physician preferences towards treatment modalities in all stages of HCC, though intermediate stage HCC was underrepresented. The studies revealed a large scope of potential attributes that may be important to patients and that many patients are willing to forgo survival to maintain quality of life. Further research should explore both preference elicitation of currently available and emerging therapies for HCC as well as the use of this data to develop patient-facing tools to assist in navigating treatment options.

### Electronic supplementary material

Below is the link to the electronic supplementary material.

Supplementary Material 1

## Data Availability

No datasets were generated or analysed during the current study.
