# Supplementary material for: Scoping review of values elicitation tools for treatment decisions in hepatocellular carcinoma
Source: BMC Gastroenterol. 2024 Feb 28;24:90. doi: 10.1186/s12876-024-03167-1 (PMC10900684; doi:10.1186/s12876-024-03167-1)
Supplement: Supplementary file 1 — Supplementary Material 1 [file 12876_2024_3167_MOESM1_ESM.docx]

**Appendix A.** Full search strategy for scoping review

| PubMed | | |
| --- | --- | --- |
| Search # | Query | Results |
| #1 | “Liver neoplasms”[mesh] OR Liver Neoplasm[tiab] OR Hepatic Neoplasms[tiab] OR Hepatic Neoplasm[tiab] OR Cancer of Liver[tiab] OR Hepatocellular Cancer[tiab] OR Hepatocellular Cancers[tiab] OR Hepatic Cancer[tiab] OR Hepatic Cancers[tiab] OR Liver Cancer[tiab] OR Liver Cancers[tiab] OR Cancer of the Liver[tiab] OR "Carcinoma, Hepatocellular"[Mesh] OR "hepatocellular carcinoma"[tiab] OR "hepatocellular carcinomas"[tiab] OR "HCC"[tiab] OR "liver cell carcinoma"[tiab] OR "liver cell carcinomas"[tiab] OR hepatoma[tiab] OR hepatomas[tiab] OR "hepatic cell carcinoma"[tiab] OR "hepatic cell carcinomas"[tiab] OR "liver carcinoma"[tiab] OR "liver carcinomas"[tiab] OR "hepatic carcinoma"[tiab] OR "hepatic carcinomas"[tiab] OR hepatocarcinoma[tiab] OR hepatocarcinomas[tiab] | 244,128 |
| #2 | (patient preference[mesh] OR decision making[mesh] OR patient preference*[tiab] OR patient perception*[tiab] OR patient choice*[tiab] OR patient perspective*[tiab] OR patient rank*[tiab] OR patient rating*[tiab] OR patient rated[tiab] OR patient weighting*[tiab] OR patient elicitation[tiab] OR patient priorit*[tiab] OR caregiver preference*[tiab] OR caregiver perception*[tiab] OR caregiver choice*[tiab] OR caregiver perspective*[tiab] OR caregiver rating[tiab] OR caregiver rated[tiab] OR caregiver priorit*[tiab] OR provider preference*[tiab] OR provider perception*[tiab] OR provider choice*[tiab] OR provider perspective*[tiab] OR provider rank*[tiab] OR provider rating[tiab] OR provider rated[tiab] OR provider elicitation[tiab] OR provider priorit*[tiab] OR clinician preference*[tiab] OR clinician perception*[tiab] OR clinician choice*[tiab] OR clinician perspective*[tiab] OR clinician rank*[tiab] OR clinician rating[tiab] OR clinician rated[tiab] OR clinician priorit*[tiab] OR stakeholder preference*[tiab] OR stakeholder perception*[tiab] OR stakeholder choice*[tiab] OR stakeholder perspective*[tiab] OR stakeholder rank*[tiab] OR stakeholder elicitation[tiab] OR stakeholder priorit*[tiab] OR preference elicitation[tiab] OR personal perspective elicitation[tiab] OR direct elicitation[tiab] OR stated preference*[tiab] OR stated choice*[tiab] OR choice experiment*[tiab] OR preference-based approach*[tiab] OR preference-based method*[tiab] OR conjoint analys*[tiab] OR choice model*[tiab] OR tradeoff*[tiab] OR trade-off*[tiab] OR best-worst[tiab] OR maximum difference scaling[tiab] OR maxdiff[tiab] OR max-diff[tiab] OR discrete choice[tiab] OR sawtooth[tiab] OR point allocation[tiab] OR magnitude estimation[tiab] OR pairwise comparison[tiab] OR paired comparison[tiab] OR budget allocation[tiab] OR willingness to pay[tiab] OR willingness to accept[tiab] OR contingent valuation[tiab] OR standard gamble[tiab] OR direct assessment[tiab] OR direct preference[tiab] OR part-worth utilities[tiab] OR self-explicated[tiab]) | 318,226 |
| #3 | #1 AND #2 | 571 |

| Embase | | |
| --- | --- | --- |
| Search # | Query | Results |
| #1 | (‘liver cancer’/exp OR ‘liver cell carcinoma’/exp OR (‘liver cancer’ OR ‘liver cell carcinoma’ OR ‘liver cell carcinomas’ OR ‘hepatocellular carcinoma’ OR ‘hepatocellular carcinomas’ OR ‘HCC’ OR hepatoma OR hepatomas OR ‘hepatic cell carcinoma’ OR ‘hepatic cell carcinomas’ OR ‘liver carcinoma’ OR ‘liver carcinomas’ OR ‘hepatic carcinoma’ OR ‘hepatic carcinomas’ OR ‘hepatocarcinoma’ OR ‘hepatocarcinomas’):ab,ti) | 349,953 |
| #2 | ('patient preference'/exp OR 'consumer attitude'/exp) OR ((patient* OR caregiver* OR consumer* OR provider* OR clinician*) NEAR/3 (preference* OR priority OR priorities OR perspective* OR perception* OR rating* OR rated OR ranking*)) OR (preference-elicitation OR personal-perspective-elicitation OR direct-elicitation OR stated-preference* OR stated-choice* OR choice-experiment* OR preference-based-approach* OR preference-based-method* OR conjoint-analys* OR choice-model* OR tradeoff* OR trade-off* OR best-worst OR maximum-difference-scaling OR maxdiff OR max-diff OR discrete-choice OR sawtooth OR point-allocation OR magnitude-estimation OR pairwise-comparison OR paired-comparison OR budget-allocation OR willingness-to-pay OR willingness-to-accept OR contingent-valuation OR standard-gamble OR direct-assessment OR direct-preference OR part-worth-utilities OR self-explicated):ab,ti | 222,853 |
| #3 | #1 AND #2 | 898 |

| Scopus | | |
| --- | --- | --- |
| Search # | Query | Results |
| #1 | TITLE-ABS(liver-cancer OR liver-cell-carcinoma OR liver-cell-carcinomas OR hepatocellular-carcinoma OR hepatocellular-carcinomas OR HCC OR hepatoma OR hepatomas OR hepatic-cell-carcinoma OR hepatic-cell-carcinomas OR liver-carcinoma OR liver-carcinomas OR hepatic-carcinoma OR hepatic-carcinomas OR hepatocarcinoma OR hepatocarcinomas) | 183,136 |
| #2 | TITLE-ABS((patient* OR caregiver* OR consumer* OR provider* OR clinician*) W/3 (preference* OR priority OR priorities OR perspective* OR perception* OR rating* OR rated OR ranking*)) OR (preference-elicitation OR personal-perspective-elicitation OR direct-elicitation OR stated-preference* OR stated-choice* OR choice-experiment* OR preference-based-approach* OR preference-based-method* OR conjoint-analys* OR choice-model* OR tradeoff* OR trade-off* OR best-worst OR maximum-difference-scaling OR maxdiff OR max-diff OR discrete-choice OR sawtooth OR point-allocation OR magnitude-estimation OR pairwise-comparison OR paired-comparison OR budget-allocation OR willingness-to-pay OR willingness-to-accept OR contingent-valuation OR standard-gamble OR direct-assessment OR direct-preference OR part-worth-utilities OR self-explicated) | 488,652 |
| #3 | #1 AND #2 | 395 |

| Cochrane Library | | |
| --- | --- | --- |
| Search # | Query | Results |
| #1 | ((liver-cancer OR liver-cell-carcinoma OR liver-cell-carcinomas OR hepatocellular-carcinoma OR hepatocellular-carcinomas OR HCC OR hepatoma OR hepatomas OR hepatic-cell-carcinoma OR hepatic-cell-carcinomas OR liver-carcinoma OR liver-carcinomas OR hepatic-carcinoma OR hepatic-carcinomas OR hepatocarcinoma OR hepatocarcinomas)):ti,ab,kw | 7,084 |
| #2 | ((patient* OR caregiver* OR consumer* OR provider* OR clinician*) NEAR/3 (preference* OR priority OR priorities OR perspective* OR perception* OR rating* OR rated OR ranking*)) OR (preference-elicitation OR personal-perspective-elicitation OR direct-elicitation OR stated-preference* OR stated-choice* OR choice-experiment* OR preference-based-approach* OR preference-based-method* OR conjoint-analys* OR choice-model* OR tradeoff* OR trade-off* OR best-worst OR maximum-difference-scaling OR maxdiff OR max-diff OR discrete-choice OR sawtooth OR point-allocation OR magnitude-estimation OR pairwise-comparison OR paired-comparison OR budget-allocation OR willingness-to-pay OR willingness-to-accept OR contingent-valuation OR standard-gamble OR direct-assessment OR direct-preference OR part-worth-utilities OR self-explicated) | 33516 |
| #3 | #1 AND #2 | 117 |
